# Therapeutic activity of a *Saccharomyces cerevisiae*-based probiotic and inactivated whole yeast on vaginal candidiasis

**DOI:** 10.1080/21505594.2016.1213937

**Published:** 2016-07-19

**Authors:** Eva Pericolini, Elena Gabrielli, Nathalie Ballet, Samuele Sabbatini, Elena Roselletti, Amélie Cayzeele Decherf, Fanny Pélerin, Eugenio Luciano, Stefano Perito, Peter Jüsten, Anna Vecchiarelli

**Affiliations:** a Department of Experimental Medicine, Microbiology Section, University of Perugia, Perugia, Italy; b Lesaffre International, Lesaffre Group, Marcq-en-Baroeul, France; c Lesaffre Human Care, Lesaffre Group, Marcq-en-Baroeul, France

**Keywords:** *Candida albicans*, fungi, probiotics, *Saccharomyces cerevisiae*, therapeutic activity, vaginal candidiasis, vaginal health, vaginal infections

## Abstract

Vulvovaginal candidiasis is the most prevalent vaginal infection worldwide and *Candida albicans* is its major agent. Vulvovaginal candidiasis is characterized by disruption of the vaginal microbiota composition, as happens following large spectrum antibiotic usage. Recent studies support the effectiveness of oral and local probiotic treatment for prevention of recurrent vulvovaginal candidiasis. *Saccharomyces cerevisiae* is a safe yeast used as, or for, the production of ingredients for human nutrition and health. Here, we demonstrate that vaginal administration of probiotic *Saccharomyces cerevisiae* live yeast (GI) and, in part, inactivated whole yeast *Saccharomyces cerevisiae* (IY), used as post-challenge therapeutics, was able to positively influence the course of vaginal candidiasis by accelerating the clearance of the fungus. This effect was likely due to multiple interactions of *Saccharomyces cerevisiae* with *Candida albicans*. Both live and inactivated yeasts induced coaggregation of *Candida* and consequently inhibited its adherence to epithelial cells. However, only the probiotic yeast was able to suppress some major virulence factors of *Candida albicans* such as the ability to switch from yeast to mycelial form and the capacity to express several aspartyl proteases. The effectiveness of live yeast was higher than that of inactivated whole yeast suggesting that the synergy between mechanical effects and biological effects were dominant over purely mechanical effects. The protection of epithelial cells to *Candida*-induced damage was also observed. Overall, our data show for the first time that *Saccharomyces cerevisiae*-based ingredients, particularly the living cells, can exert beneficial therapeutic effects on a widespread vaginal mucosal infection.

## Introduction

Probiotics are live microorganisms that provide health benefits to the host when ingested in adequate amounts. The strains most frequently used as probiotics include: *Bifidobacterium, Lactobacillus* and *Saccharomyces* spp.^1^. Compelling evidence shows that probiotics can influence the metabolic processes of pathogens which lead to infection, and thus confer some type of protection against disease.^2-4^ In particular, it has been well established that several pathologic processes such as obesity and metabolic syndrome are associated with changes in intestinal microbiota.^5-7^ Moreover, among the health promoting properties such as antitumoral, antimicrobial and hypocholesterolemic effects have been associated with consumption of milk fermented with probiotics.^8^ Additionally, fermented milk effects have been associated with modulation of brain activity.^9^ Immunomodulatory activities of probiotics are important for control of infections and have been detected in various tissues and organs. Moreover, recent data suggest that oral administration of *Lactobacillus* (*L*.) *rhamnosus* inhibited allergen-induced airway inflammation in an experimental system of allergic asthma.^10^ Furthermore some probiotics such as *L. plantarum* can induce complex immune responses in dendritic cells^11^ which may critically impact microbe-host interactions. Probiotics have also been shown to strongly influence inflammatory responses. In addition, the administration of probiotics plays an important role in the maintenance of the epithelial barrier via control of inflammation and cell recruitment.^4^

Moreover, several studies have indicated that non-viable material of microbial origin positively affect human/animal health.^12^

*Candida albicans* (*C. albicans*) is a commensal organism that lives as a benign component of the microflora of the human oral, gastrointestinal and vaginal tracts. This fungus can shift from a commensal to a pathogenic state in response to environmental stimuli that trigger developmental programs that induce the expression of virulence factors, among which the secretion of enzymes, particularly aspartyl proteinases, affecting host immunity, plays a fundamental role.^13,14^ About 3-quarters of women during their reproductive age have at least one episode of vulvovaginal candidiasis (VVC) and approximately 6–8% of these subjects undergo recurrences (3 or more per year, RVVC).^15^ In this pathology, the vaginal microbiome has been suggested to play an important role.^16^ In fact, an abnormal vaginal microbiome is often associated with symptomatic infections such as VVC. The use of wide spectrum antibiotics leads to disruption of microflora and this condition favors *Candida* overgrowth, specifically, colonization with *C. albicans* increases approximately from 10% to 30%.^16^ However, few studies support the effectiveness of oral and local probiotics treatment with different species of Lactobacilli for prevention or therapy of recurrent RVVC,^17^ sometimes in association with antifungal drugs such as fluconazole (FLZ).^18^

With the above background, the aim of this study was to analyze the role of probiotic *Saccharomyces cerevisiae* (*S. cerevisiae*) live yeast (GI) and inactivated whole yeast *S. cerevisiae* (IY) in treating vaginal candidiasis, using a suitable “*in vivo*” experimental model. Our studies also provide some mechanistic insights into the capacity of *S. cerevisiae*-based ingredients to control this experimental vaginal infection.

## Results

### Anticandidal activity of yeast ingredients

**Figure 1.**
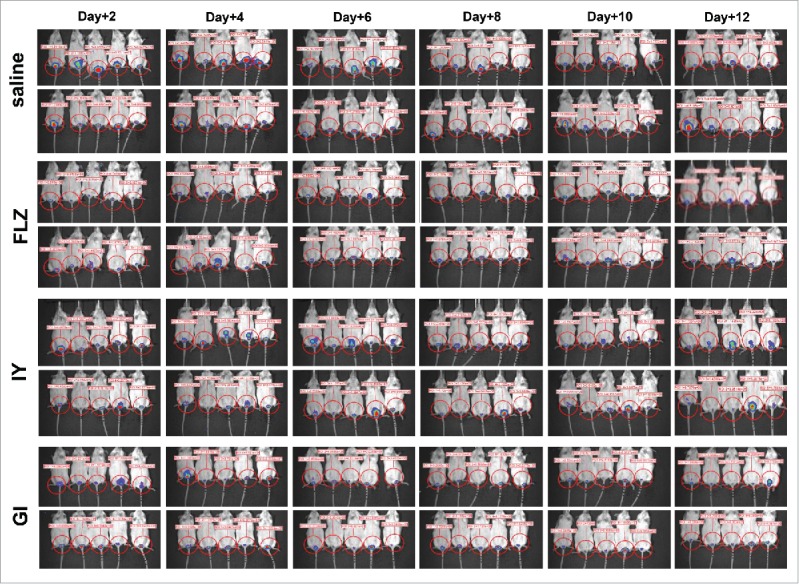
*In vivo* imaging of mice vaginally infected with BLI *Candida* and treated with a single dose of FLZ, IY or GI. Mice under pseudoestrus condition were treated intravaginally with 10 μl of saline, FLZ (200 μg/ml, 10 μl/mouse) or different yeast products: IY (100 mg/ml, 10 μl/mouse) and GI (10 mg/ml, 10 μl/mouse), 1 day after challenge (2 × 10^7^ BLI *Candida* cells/10 μl/mouse). After 2, 4, 6, 8, 10 and 12 d post-infection mice were treated intravaginally with 10 μl of coelenterazine (0.5 mg/ml) and imaged in the IVIS-200TM imaging system under anesthesia with 2.5% isoflurane. Total photon flux emission from vaginal areas within the images (Region Of Interest, ROI) of each mouse was quantified with Living ImageR software package.

**Figure 2.**
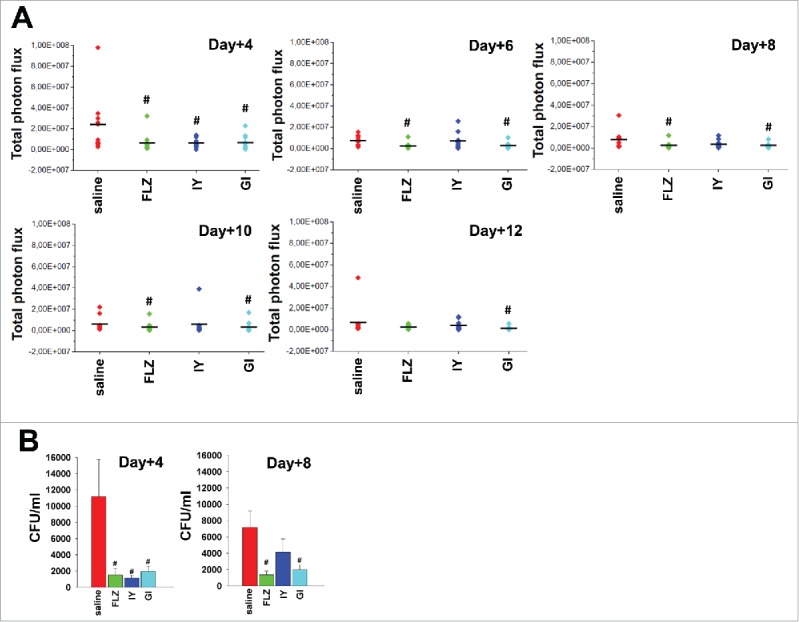
Quantification of Total photon flux emission and CFU count. Mice under pseudoestrus condition were treated intravaginally with 10 μl of saline, FLZ (200 μg/ml, 10 μl/mouse) or different yeast products: IY (100 mg/ml, 10 μl/mouse) and GI (10 mg/ml, 10 μl/mouse), 1 day after challenge (2 × 10^7^ BLI *Candida* cells/10 μl/mouse). After 4, 6, 8, 10 and 12 d post-infection mice were treated intravaginally with 10 μl of coelenterazine (0.5 mg/ml) and imaged in the IVIS-200TM imaging system under anesthesia with 2.5% isoflurane. Total photon flux emission from vaginal areas within the images (Region Of Interest, ROI) of each mouse was quantified with Living ImageR software package. Quantification of Total photon flux emission from ROI (n = 10 mice for each group from 2 different experiments) was evaluated and the statistical significance was determined with Mann-Whitney U-test (A). Values of *p* < 0.05 were considered significant (FLZ- or yeast products-treated infected mice *vs* saline-treated infected mice). In selected experiments, after 4 and 8 d post-infection the CFU in vaginal washes of the mice (n = 6 mice for each group from 2 different experiments) were evaluated and the statistical significance was determined with Mann-Whitney U-test (B). Data are expressed as mean ± SEM. Values of *p* < 0.05 were considered significant (FLZ- or yeast products-treated infected mice *vs* saline-treated infected mice).

Previous studies show that the administration of live yeast *S. cerevisiae* may be beneficially used in a variety of pathologies.^4^ Here we analyzed the effect of administration of *S. cerevisiae* live yeast (GI) and inactivated whole yeast *S. cerevisiae* (IY) on the course of vaginal candidiasis in a mouse experimental model by using bioluminescent *C. albicans* (BLI *Candida*).^19^ To this end following preliminary dose-findings experiments *in vivo*, mice under pseudoestrus conditions were treated intravaginally with IY (1 mg/10µl/mouse) and GI (0.1 mg/10µl/mouse) 1 day after intravaginal challenge with BLI *Candida* (10µl/mouse of 2 × 10^9^/ml BLI *Candida* suspension). Saline-treated and FLZ-treated mice served as negative and positive controls, respectively. The results reported in Figure 1, panel A of Figure 2 and Figure S1 show that a significant reduction of fungal load was observed 4 d after infection in mice treated with both IY and GI. The effect of GI was evidenced until 12 d post-infection, while the effect of IY was only observed until day 4. These results, obtained by measurement of bioluminescence, were confirmed by colony forming units (CFU) recovery from vaginal washes (panel B of Fig. 2). Noteworthy, on day +4 post-challenge, the anti-*Candida* effect of IY and GI compared with FLZ (panel B of Fig. 2).

**Figure 3.**
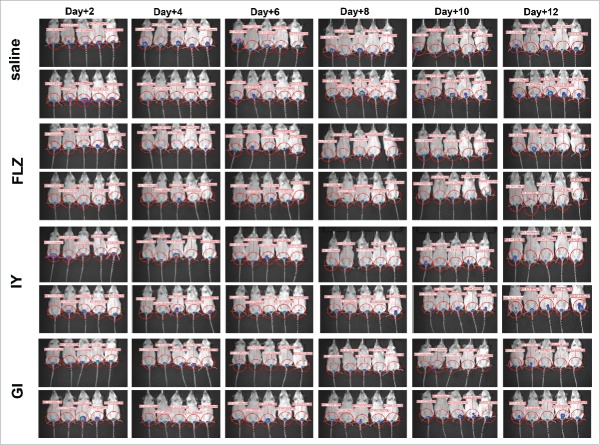
*In vivo* imaging of mice vaginally infected with BLI *Candida* and treated daily with FLZ, IY or GI. Mice under pseudoestrus condition were treated intravaginally with 10 μl of saline, FLZ (200 μg/ml, 10 μl/mouse) or different yeast products: IY (100 mg/ml, 10 μl/mouse) and GI (10 mg/ml, 10 μl/mouse), every day starting from day +1 after challenge (2 × 10^7^ BLI *Candida* cells/10 μl/mouse). After 2, 4, 6, 8, 10 and 12 d post-infection mice were treated intravaginally with 10 μl of coelenterazine (0.5 mg/ml) and imaged in the IVIS-200TM imaging system under anesthesia with 2.5% isoflurane. Total photon flux emission from vaginal areas within the images (Region Of Interest, ROI) of each mouse was quantified with Living ImageR software package.

**Figure 4.**
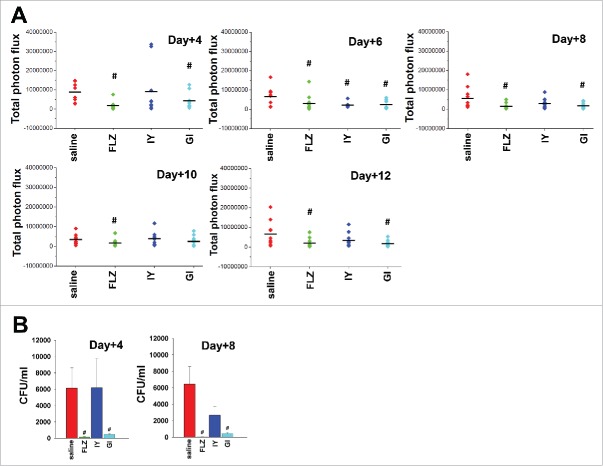
Quantification of Total photon flux emission and CFU count. Mice under pseudoestrus condition were treated intravaginally with 10 μl of saline, FLZ (200 μg/ml, 10 μl/mouse) or different yeast products: IY (100 mg/ml, 10 μl/mouse) and GI (10 mg/ml, 10 μl/mouse), every day starting from day +1 after challenge (2 × 10^7^ BLI *Candida* cells/10 μl/mouse). After 4, 6, 8, 10 and 12 d post-infection mice were treated intravaginally with 10 μl of coelenterazine (0.5 mg/ml) and imaged in the IVIS-200TM imaging system under anesthesia with 2.5% isoflurane. Total photon flux emission from vaginal areas within the images (Region Of Interest, ROI) of each mouse was quantified with Living ImageR software package. Quantification of Total photon flux emission from ROI (n = 10 mice for each group from 2 different experiments) was evaluated and the statistical significance was determined with Mann-Whitney U-test (A). Values of *p* < 0.05 were considered significant (FLZ- or yeast products-treated infected mice *vs* saline-treated infected mice). In selected experiments, after 4 and 8 d post-infection the CFU in vaginal washes of the mice (n = 6 mice for each group from 2 different experiments) were evaluated and the statistical significance was determined with Mann-Whitney U-test (B). Data are expressed as mean ± SEM. Values of *p* <0.05 were considered significant (FLZ- or yeast products-treated infected mice *vs* saline-treated infected mice).

To evaluate whether daily administration of IY and GI could enhance the anti-*Candida* beneficial effect of both products, we treated the mice with the same dose above every day starting from day +1 post challenge until day 12. The results reported in Figure 3, panel A of Figure 4 and Figure S2 show that this schedule of administration confirm that GI displayed a curative effect until day +12 post-infection, while the effect of IY was only minimally extended to 6 d post-infection. These results were obtained by using the measurement of bioluminescence of the vaginal region in live animals as well as by determination of CFU from vaginal washes (panel B of Fig. 4). In these experiments, GI compared well with FLZ in the anticandidal activity, both on day +4 and day +8 (panel B of Fig. 4). While the CFU level was numerically lower with IY on day +8, showing 42.5% of CFU inhibition, the difference was not significant.

## Mechanistic insights into the observed anticandidal activity of yeast ingredients

### Aggregation and adherence

**Figure 5.**
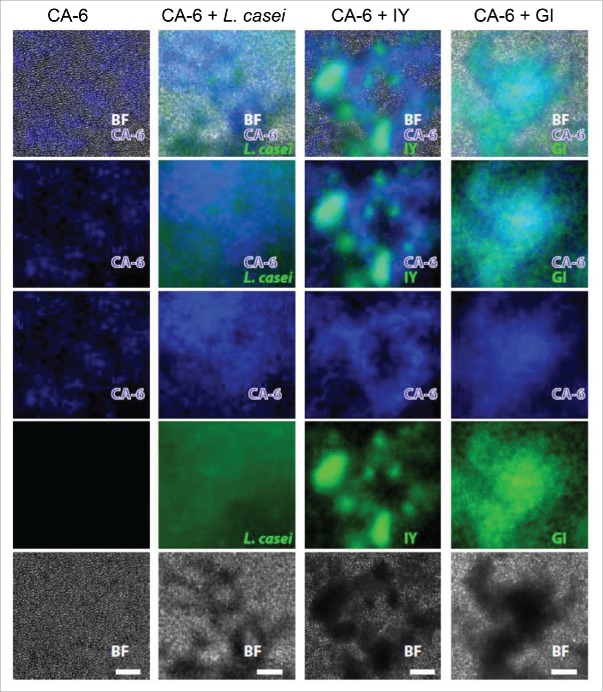
*Candida* aggregation by FITC-*L. casei*, FITC-IY and FITC-GI. CFW-CA-6 (1 × 10^9^/ml) in 500 µl of PBS was mixed or not with equal volume of FITC-*L. casei* (3.3 McFarland), FITC-IY or FITC-GI (both 10 mg/ml). Then the samples were vortexed for 10 sec and incubated for 4 h at 37°C under agitation. The suspensions were observed and photographed by fluorescent light microscopy. Images are representative of 3 separate experiments with similar results. (Scale Bar = 50 µm, Magnification 20x). BF = bright field; CA-6 = blue; *L. casei*, IY and GI = green.

**Figure 6.**
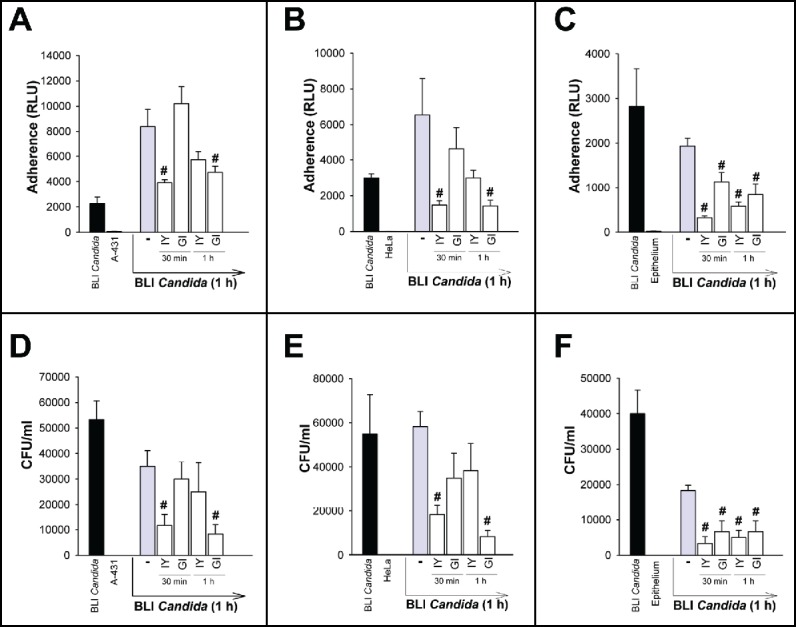
Effect of IY and GI on BLI *Candida* adherence and growth. A-431 cells (panels A and D), HeLa cells (panels B and E) or vaginal epithelium (panels C and F) were incubated for 30 min or 1 h in the presence or absence of IY or GI (both 100 mg/ml). After incubation, cells were washed 5 times, then incubated in the presence or absence of BLI *Candida* for 1 h. After incubation, cells were washed 5 times, then bioluminescence emission was evaluated by luminometer after adding coelentarazine (panels A-C). Trypsin/EDTA solution (100 µl) was added in each well, then the plates were incubated for 15 min at 37°C to dissociate cells. Hence, the cellular suspension was diluted (1/10), plated onto CHROMagar Candida and incubated at 37°C for 48 h. The fungal load (the adherent BLI *Candida*) was quantified as the number of CFU/ml (panels D-F). BLI *Candida* refers to *Candida* adhesion to empty wells (black bars). Data are expressed as mean ± SEM. #, *p* < 0.05 cells incubated with BLI *Candida* plus IY or GI *vs* cells incubated with BLI *Candida* alone.

It has been shown that some microorganisms, such as *L.* spp. used successfully to prevent vaginal recurrent infections, could exert strong adhesion forces with pathogens and bind the strains into aggregates.^20^ In this line, we analyzed whether IY and GI were able to induce *C. albicans* aggregation. Positive control such as *L. casei*^21^ was also used. The results reported in Table 1 show that IY is able to self-aggregate with the maximum score (score = 4) (according to Verdenelli M.C. et al.^21^). The same maximum score was observed when IY was added with *C. albicans*. Conversely, GI is unable to self-aggregate, but when mixed with *C. albicans*, caused a consistent fungal aggregation (score = 3). A representative image is reported in Figure 5. Given that pathogen aggregation may compromise its capacity to adhere to epithelium, we tested this possibility in experiments of BLI *Candida* adherence to various kinds of epithelial cells. To this end IY and GI were added to vaginal epithelial cell line A-431 or cervical cancer cell line HeLa or human vaginal epithelium for 30 min or 1 h, then BLI *Candida* was added for 1 h. The bioluminescence emission of adhered BLI *Candida* was evaluated. Results reported in panels A-C of Figure 6 show that treatment with IY and GI for 30 min and 1 h significantly prevented the adherence of BLI *Candida* to the vaginal epithelium (Fig. 6C). Regarding the effect on A-431 and HeLa cells, a sporadic inhibition of adherence was manifested. In particular, the effect of IY was observed after 30 min of pretreatment, while the effect of GI was evident after 1 h, in both cell lines (Fig. 6A and B). Determination of inhibition of *Candida* adherence was also performed by using CFU. The results obtained (Fig. 6 panels D–F) further suggested that *Candida* coaggregation, induced by IY and GI, is involved in adherence inhibition of the fungus to vaginal epithelial cells.

**Table 1.** Coaggregation between *C. albicans* (CA-6) (1 × 10^9^/ml) and *L. casei* (3.3 McFarland) or IY and GI (both 10 mg/ml). Scores from 0 (no aggregation) to 4 (maximum aggregation) and mean score are shown. Data are from replicates samples of 6 different experiments.

	CA-6	*L. casei*	IY	GI	CA-6 + *L. casei*	CA-6 + IY	CA-6 + GI
Exp. 1	0	0	4	0	3	4	3
Exp. 2	0	0	4	0	3	4	3
Exp. 3	0	0	4	0	3	4	3
Exp. 4	0	0	4	0	3	4	3
Exp. 5	0	0	4	0	3	4	3
Exp. 6	0	0	4	0	3	4	3
**Mean score**	**0**	**0**	**4**	**0**	**3**	**4**	**3**

**Figure 7.**
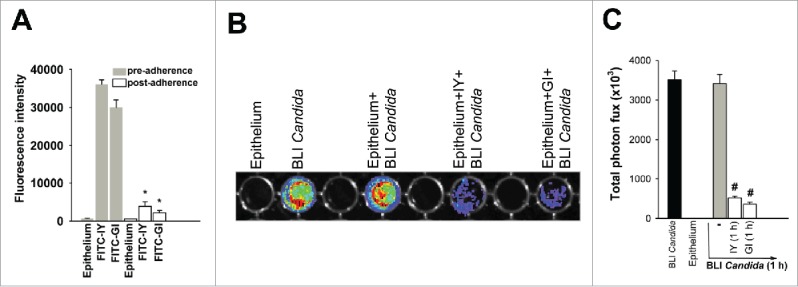
Quantification of FITC-IY and FITC-GI adherence on vaginal epithelium and *in vitro* imaging of BLI *Candida* adherence. 100 µl of FITC-IY or FITC-GI (both 100 mg/ml) were added to vaginal epithelium grown in black 96-well microtiter plates with a transparent bottom and analyzed for fluorescence signals (λ = 600 nm) (pre-adherence), then incubated for 1 h at 37°C plus 5% CO_2_. After incubation, cells were extensively washed 5 times with PBS and then analyzed again for fluorescence signals (λ = 600 nm) (post-adherence) (A). Data are expressed as mean ± SEM of triplicate samples of 3 different experiments. *, *p* < 0.05 FITC-IY-and FITC-GI-treated vaginal epithelium *vs* untreated. Vaginal epithelium was grown in black 96-well microtiter plates with a transparent bottom (100 µl/well). Vaginal epithelium were then incubated in the presence or absence of 100 µl of IY or GI (both 100 mg/ml) for 1 h at 37°C plus 5% CO_2_, extensively washed with PBS and then incubated with 100 µl of BLI *Candida* (1 × 10^6^/ml) for 1 h. After co-incubation, non-adherent BLI *Candida* cells were extensively washed with PBS and 100 μl of 2 μM coelenterazine in LA buffer were added in each well. Luciferase activity was measured by using IVIS-200TM imaging system (Xenogen Inc.) (B). Total photon flux emission from each well (Region Of Interest, ROI) was quantified with Living ImageR software package (C). Data are expressed as mean ± SEM of duplicate samples of 3 different experiments. #, *p* < 0.05 IY or GI plus BLI *Candida*-treated vaginal epithelium *vs* BLI *Candida*-treated vaginal epithelium.

The data above could be explained by competition for *C. albicans* adherence exerted by the live yeast cells or the inactivated ones. To verify this hypothesis, human vaginal epithelium was incubated with 100 µl of FITC-IY or FITC-GI (both 100 mg/ml) and immediately analyzed for fluorescence signals. The fluorescence intensity was 3.6 × 10^4^ ± 1168 and 3 × 10^4^ ± 1978 for both IY and GI, respectively (Fig. 7A). Then the cultures were incubated for 1 h at 37°C and 5% CO_2_. After incubation, cells were extensively washed with phosphate-buffered saline (PBS) and analyzed for fluorescence intensity. The results reported in Figure 7A show that fluorescence signal was detected for both IY and GI, proving their ability to adhere to the vaginal epithelium. Then, in parallel experiment, IY and GI were added to epithelium for 1 h, washed, then BLI *Candida* was added for 1 h and washed again. A representative image of BLI *Candida* adherence on vaginal epithelium is shown (Fig. 7B). Total photon flux emission from 3 different experiments is shown in Figure 7C. These results show that both IY and GI significantly reduce BLI *Candida* adherence on vaginal epithelium. Overall, the data of the above experiments suggest for a direct competition for *C. albicans* adherence by the yeasts.

Finally, we evaluated whether live *Saccharomyces* cells (GI) were able to grow and multiply in the presence of vaginal epithelium. CFU were performed after 1 h and 18 h of incubation and the results showed that under our experimental conditions GI did not undergo significant proliferation neither after 1 h nor after 18 h of incubation (not shown).

### Germ-tube and hyphae formation

**Figure 8.**
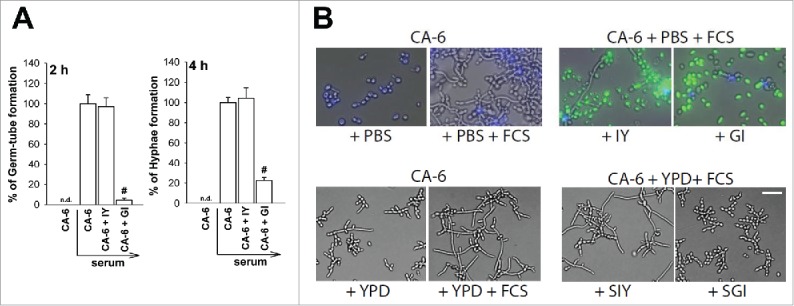
Germ-tube and hyphae formation. Germ-tube and hyphae formation of *C. albicans* (CA-6) (1 × 10^6^/ml) in PBS or PBS plus serum in the presence or absence of IY and GI (both 10 mg/ml) are shown (A). Data are reported as the percentage of germ-tube and hyphae formation compared to control (CA-6 treated with serum) Data are the mean ± SEM of 3 different experiments. #, *p* < 0.05 GI-treated CA-6 plus serum *vs* CA-6 plus serum. Hyphae formation of CFW-CA-6 (1 × 10^6^/ml) in PBS or PBS plus serum in the presence or absence of FITC-IY, FITC-GI (both 10 mg/ml) is shown (B, upper panels). Hyphae formation of CA-6 (1 × 10^6^/ml) in YPD or YPD plus serum in the presence or absence of cell-free supernatants of IY and GI (both 2 mg/ml) (SIY and SGI) in YPD medium, is shown (B, lower panels). Images are representative of 3 separate experiments with similar results. (Scale Bar = 10 µm, Magnification 20x). CA-6 = blue; IY and GI = green

*C. albicans* undergoes a yeast to hyphae transition when cultured in the presence of serum. In our experimental system we mixed *C. albicans* in PBS plus 10% FCS with IY and GI (both 10 mg/ml). After 2 or 4 h, germ-tube and hyphae formation were evaluated. The results reported in Figure 8A show that IY did neither inhibit nor increase *C. albicans* transition to germ-tube and hyphae. Conversely, a strong inhibition of germ-tube and hyphae formation was caused by GI. Moreover, results in Figure 8B clearly show that both IY and GI stick to *C. albicans* but the inhibition of hyphae formation was observed only when *Candida* was incubated with GI. Cell-free supernatant of GI cultures, but not IY, was also able to inhibit hyphae formation suggesting that the effect of GI could be mediated not only by the physically interaction of GI with *Candida* but also by the release of soluble factors.

### Inhibition of *SAP2* and *SAP6* expression

**Figure 9.**
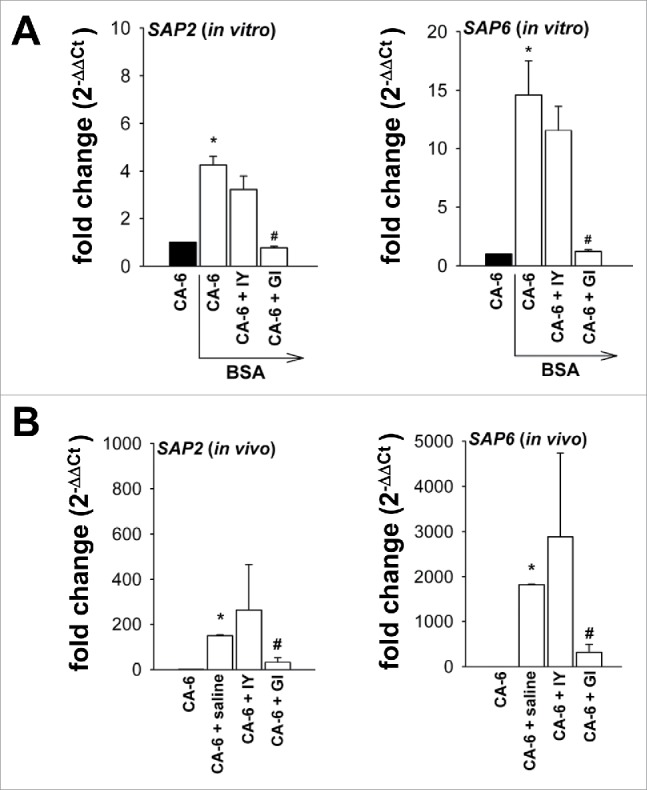
Quantitative analysis of *SAP2* and *SAP6* genes expression. CA-6 (2 × 10^6^/ml) was incubated in YEPD medium or YEPD medium plus 1% BSA in the presence or absence of IY or GI (both 10 mg/ml) for 24 h at 37°C under agitation. After incubation, fungal cells were lysed and total RNA was extracted and retro-transcribed in cDNA. *C. albicans ACT1, SAP2* and *SAP6* genes were detected by real-time qPCR and cDNA quantities were reported as fold changes relative to the *Candida* alone. Black bars indicate CA-6 in YEPD medium without BSA. Data show the mean ± SEM of triplicates of 4 different experiments (A).*, *p* < 0.05 CA-6 plus BSA *vs* CA-6. #, *p* < 0.05 GI-treated CA-6 plus BSA *vs* CA-6 plus BSA. Vaginal washes from mice intravaginally infected with 2 × 10^7^ CA-6 cells/10 µl/mouse and daily treated with IY (100 mg/ml) or GI (10 mg/ml), were obtained 8 d post-infection. Vaginal washes were centrifuged at 3000 rpm for 5 min, then cellular fractions were lysed and total RNA was extracted and retro-transcribed in cDNA. *C. albicans ACT1, SAP2* and *SAP6* genes were detected by real-time PCR and cDNA quantities were reported as 2^−ΔΔCT^ relative to the *Candida* suspension alone. Data show the mean ± SEM of triplicates samples of 3 different mice (B).*, *p* < 0.05 saline-treated infected mice *vs* CA-6 suspension used for infection. #,*p* < 0.05 GI-treated infected mice *vs* saline-treated infected mice.

Secretory aspartyl proteinases (Sap) have been shown to act as major pathogenicity determinants in vaginal candidiasis.^13,14^ In this pathology, clinical and experimental observations revealed that a robust vaginal inflammatory response is a hallmark of the pathogenic process^22^ and that Sap2 and Sap6 actively participate in inflammation.^23^ Thus we tested whether IY or GI were able to affect the expression of these 2 Sap. To this end *C. albicans* cells were incubated with BSA, a classical *SAP* inducer,^24^ in the presence or absence of IY and GI. After 24 h of incubation, *SAP* expression was evaluated by quantitative (q) RT-PCR. The results reported in Figure 9A show that *SAP2* and *SAP6* are, as expected, upregulated upon BSA induction. Noteworthy GI, but not IY, was able to significantly reduce the expression of both *SAP*. Moreover, *SAP* gene expression was also evaluated in vaginal washes of mice, treated daily with saline (10 μl/mouse), IY (100 mg/ml, 10 μl/mouse) or GI (10 mg/ml, 10 μl/mouse), after 8 d of *Candida* infection. Results reported in panel B of Figure 9 show that GI, but not IY, was able to strongly reduce the expression of both *SAP2* and *SAP6* also in an “*in vivo”* experimental model.

### Cell damage

**Figure 10.**
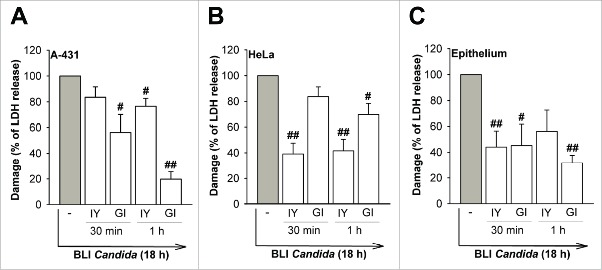
Effect of IY and GI on cellular damage induced by BLI *Candida*. A-431 cells (A), HeLa cells (B) or vaginal epithelium (C) were incubated for 30 min or 1 h in the presence or absence of IY and GI (both 100 mg/ml). After incubation, cells were washed 5 times, then incubated in the presence or absence of BLI *Candida* (1 × 10^6^/ml) for 18 h. After incubation, cellular damage induced by BLI *Candida* was determined by the release of LDH. Data show the mean ± SEM of triplicates samples of 3 different experiments. Values of *p* < 0.05 and *p*< 0.01 were considered significant. #,*p* < 0.05 and ##,*p* < 0.01 cells incubated with BLI *Candida* plus GI or IY *vs* cells incubated with BLI *Candida* alone.

Finally, the capacity of both IY and GI to protect from *C. albicans-*induced damage to vaginal epithelial cells or in the vaginal epithelium was evaluated. To this end, IY and GI were added to the vaginal epithelial cell line A-431 or to the cervical cancer cell line HeLa or human vaginal epithelium for 30 min or 1 h and then BLI *Candida* was added for 18 h. The damage was measured by lactate dehydrogenase (LDH) release as previously described.^25^ The results showed that by using A-431 and HeLa cells or vaginal epithelium both IY and GI prevented the damage induced by *C. albicans* (Fig. 10). Indeed, GI shows an important and clear protective effect against *C. albicans* and damage of vaginal epithelium.

## Discussion

Probiotics have long been considered a potential alternative or integrative treatment for the control of various infectious or non-infectious pathologies. The rationale resides on the well-known influence of the microbiota on an organism's homeostasis and the potential of probiotics to reconstitute microbiota dysbiosis or losses. For example, some probiotics manifested a supportive therapeutic effect on ulcerative colitis in an experimental animal model.^26^ Moreover, probiotics are widely used as an efficient adjuvant against other gastrointestinal tract disorders such as treatment of several types of diarrhea, both in humans and animals. It has been also reported that live *S. cerevisiae* was able to significantly enhance the viability of probiotic strains such as *L. rhamnosus* under acidic conditions, this effect being related to pH, probiotic and yeast concentration.^27^ This study also provides evidence that supplementation with vaginal *L. rhamnosus* may be useful in discouraging growth of pathogenic microorganisms especially after antibiotic therapy; therefore, this treatment may be considered for preventing vaginal infections. Moreover, a considerable amount of published data indicates that the use of non-viable microbial cells or cell components can positively influence the host's immune system.^12^

With this scientific background we focused our attention on potential therapeutic activity of one probiotic i.e. live *S. cerevisiae* yeast (GI) and one inactivated yeast, obtained by drum-drying (IY), against vaginal candidiasis caused by *C. albicans*, using a well-known mouse model.^23^ In summary, our experiments showed that a post-challenge administration of the probiotic yeast and, to a lesser degree, the inactivated yeast caused a statistically significant increase of *C. albicans* clearance from the mouse vagina, meaning a consistent anti-infectious benefit. We also observed that both *S. cerevisiae* live and inactivated cells could exert some kinds of “mechanic” such as *C. albicans* aggregation or competition for space occupation on epithelial tissues, as in fungus adherence to the vaginal epithelium. However, only the probiotic yeast could cause inhibition of defined virulence traits of *C. albicans* in the vaginal setting such as *SAP* expression and yeast to hyphae transition. This suggests that the therapeutic benefit is optimally achievable only with the probiotic GI and, importantly, the benefit could be due to dampening *C. albicans* virulence. Candidal vaginitis is an acute inflammatory disease that affects many women of fertile age, with no definitive cure and with possible recurrence causing devastation of quality of life. The local inflammatory response in the vagina is considered the crucial event in sustaining pathologic processes.^22^ One of the causes identified for clinical inflammation occurs in cases of disturbed balance between the host and the colonizing microorganisms.^28^ In this study we used CD1 mouse model of vaginal candidiasis as previously described.^23,29^ Indeed the outbred mice are by definition closer to human situation. Moreover, in the most sensitive inbred C57BL/6 mice, the infection is not limited to vagina but goes upper to the uterus, causing vagina-unrelated micro- and macroscopic damage of epithelial cells quite well described in a previous paper of ours.^30^

The vaginal microbiota plays an important role in health and disease. *Lactobacillus* is the dominant genus in the vagina of healthy women whereas it is depleted during bacterial vaginosis.^31^ Several studies evaluated the effectiveness of different functional ingredients such as probiotics in preventing RVVC. In particular, recent results proved the beneficial effect of *Lactobacillus* spp. oral treatment in curing this type of infection.^32^ Other studies evaluated the effectiveness of probiotics in the restitution of normal vaginal microflora after vaginal infection including VVC.^28,31^

*Lactobacillus* spp. are an important element of vaginal microflora because their production of lactic acid keeps the vaginal pH low and prevents overgrowth of other pathogens; thus, they are the most common probiotics used in treating vaginal infections.^31,32^

*Saccharomyces cerevisiae* is non-pathogenic yeast safely used for human nutrition and health, and rarely reported to cause infection in highly predisposed, high-risk subjects.^33^ The majority of studies performed with probiotic *S. cerevisiae* strains have focused on gastrointestinal tract infections, where the microbiota unbalance is evident.^27^ However, it has been recently reported that *S. cerevisiae* enhances the survival and therapeutic potential of probiotic *L. rhamnosus*^27^ that is often used in treating vaginal infection.^34^ In this paper we demonstrate that intravaginal administration of *S. cerevisiae*-based ingredients [living cells (GI) or dead (IY)] were able to influence the course of vaginal candidiasis by accelerating the clearance of the fungus from the vagina. Noteworthy, we administered IY and GI after infection, thus the effect could be considered as a therapeutic option. Overall, the effectiveness of GI was higher than that of IY, and likely incorporating biological effects dominantly over purely mechanical effects, as it happens with IY only. In fact, GI was able to inhibit some major virulence factors of *C. albicans* such as the ability to switch from yeast to mycelial form under conditions of serum-induced hyphal differentiation, and the capacity to repress 2 important virulence factors, *SAP2* and *SAP6*. The inactivated yeast IY was able to induce *C. albicans* coaggregation and exerted some protection to epithelial cells from *Candida* induced damage. These effects are probably due to mechanical effects. In fact, the inactivated yeast was incapable of influencing virulence factors. This different behavior correlates with the less effectiveness of IY with respect to GI and points out that the probiotic yeast exerts the bigger pressure on pathogen populations.

We report here for the first time that the *S. cerevisiae*-based ingredients (living cells or dead) could be beneficial in curing some vaginal mucosal infections. It is conceivable that the different epithelial cell lines used may produce some slight different results in response to IY and GI, nonetheless we clearly demonstrate that both IY and GI show competition and interference in the adhesion of *Candida* to vaginal epithelium, that is the most reliable indicator for interaction with human vaginal epithelium. This is likely due to multiple effects including the ability of IY and GI to occupy the similar cellular receptors exploited by pathogenic *Candida* and, as a consequence, to inhibit adherence. This could be related to the possibility of *S. cerevisiae* and *Candida* sharing some external antigens (for instance the polysaccharides mannan and glucan PAMP) involved in the recognition by cellular receptors.

Coaggregation is one of the mechanisms exerted by probiotics to create a competitive micro-environment around the pathogen. Moreover, a recent exhaustive review by de Groot et al. analyzes the role of fungal adhesins in modulating adhesion, aggregation, and biofilm formation.^35^

Both IY and GI are able to induce aggregation of *Candida*. We do not have an explanation about the mechanism that induces the coaggregation, but in a previous paper it has been demonstrated that the coaggregation between vaginal Staphylococci and Lactobacilli is due to adhesive forces that occur instantly upon contact and matured within one to two minutes.^20^ Thus the mechanism could be similar to that described above.

The most relevant interaction of our probiotic GI live yeast with *C. albicans* is the ability to influence the major virulence factors such as hyphae transition and expression of *SAP*. Indeed, in our system GI (live yeast cells), differently from IY (inactivated whole yeast), was able to strongly inhibit germ-tube and hyphal formation under conditions of serum-induced hyphal differentiation. It is unclear how the live yeast inhibits hyphal growth. It may be hypothesized that this probiotic yeast influences hyphal transition by using, hence making unavailable to *Candida*, some serum components which induce hyphal morphogenesis by the stimulation of the cAMP-Ras1p signaling pathway, that plays the major role in the morphogenesis of *C. albicans*^36^ or by affecting the production of *C. albicans* quorum sensing molecules which controls filamentation in such pathogenic polymorphic fungi.^37^ In fact, competition for use of nutrients and growth stimulators can be an important mechanism of probiotic activity.

Given that morphogenesis is a key virulence factor of *C. albicans*, it could be a target for the development of antifungal drugs. Indeed, inhibiting morphogenesis has been considered for treating candidiasis.^38^

Aspartyl proteases are also major virulence factors of *C. albicans* because they are involved in adhesion of the fungus and damage of epithelial cells.^25^ *C. albicans* expresses a family of 10 *SAP* genes that are grouped as follow: *SAP1* to *SAP3, SAP4* to *SAP6, SAP7, SAP8, SAP9* and *SAP10* based upon their sequence homologies and pH activities.^13^ Each *C. albicans* Sap protein has a distinct substrate cleavage site and pH optimum. Sap1 to Sap3 and Sap8 have activity at lower pH values (2.5 to 5.0), whereas Sap4 to Sap6 have better activity at higher pH values.^13^ Sap expression levels and activities are regulated by cell morph-type and environmental stimuli. It has been reported that Sap1-Sap3 are expressed predominantly in yeast cells, whereas Sap4-Sap6 in hyphal cells, although the *in vivo* demonstration of these specific associations has never been convincingly shown. In our hands, both *in vitro* and *in vivo* in the CD1 vaginal candidiasis model, we observed that both *SAP2* and *SAP6* genes were expressed, consistently with some previous reports in human infection.^39^ Furthermore our recent observations in *C. albicans*-infected CD1 mice also coherently indicate the presence of both yeast and hyphal forms of the fungus in the infected vagina (manuscript in preparation).

**Figure 11.**
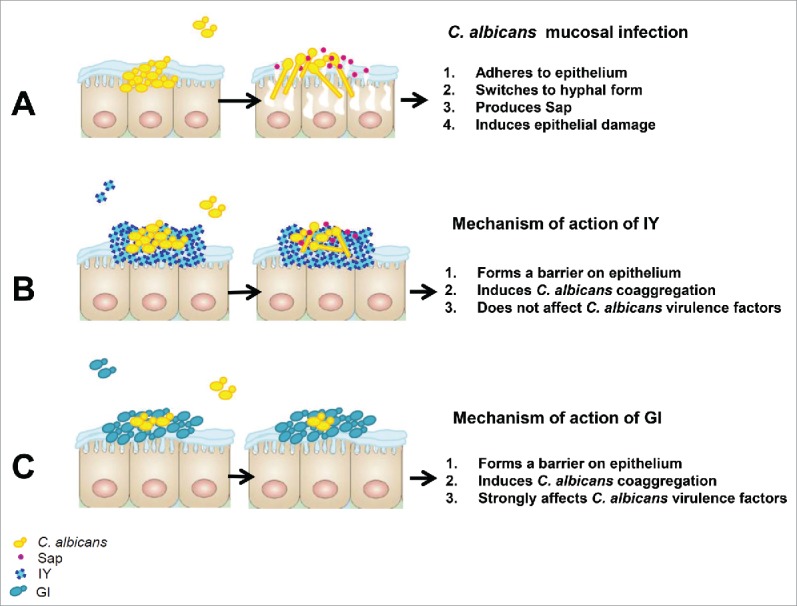
IY and GI mechanism of action on vaginal epithelium. *C. albicans* is able to cause epithelial cell damage through its capacity to adhere to epithelium, to switch from yeast to hyphal form and to produce aspartyl proteinases (Sap) (A). IY reduces epithelial damage induced by *C. albicans*, preventing *C. albicans* adherence by the formation of a barrier on epithelium and by the induction of *C. albicans* coaggregation (B). GI reduces epithelial damage induced by *C. albicans*, preventing *C. albicans* adherence by the formation of a barrier on epithelium, by the induction of *C. albicans* coaggregation and by strongly affecting *C. albicans* virulence factors (C).

We previously reported that Sap2 and Sap6 are extensively involved in the inflammatory processes, and it is well known that inflammation is strictly related to pathogenesis in vaginal candidiasis.^23,40,^^41^ As a consequence, the reduced expression of *SAP2* and *SAP6* GI-mediated could partially account for the *in vivo* protective effect. Noteworthy, a strong inhibition of *SAP2* and *SAP6* expression was observed only when GI (live yeast cells) but not IY (inactivated yeast), was used, suggesting that the inhibition of *SAP* could be related to biomolecules present in living cells. In this study we observed that both IY and GI are able to protect the integrity of epithelial cells damaged by *C. albicans*. This effect was evidenced for both preparations, but with different mechanisms. Indeed in this study we demonstrate that the beneficial effect of inactivated yeast of *S. cerevisiae* (IY) was greatly due to its ability to induce coaggregation of *C. albicans* and to inhibit adherence to vaginal epithelium. The effect of live *S. cerevisiae* cells (GI) was due to more complex mechanisms including inhibition of mycelial transition and *SAP* expression (see mechanism of action in Fig. 11).

These differences in the complexity of yeast ingredients effects directly or indirectly strictly influence the virulence of pathogen and integrity of epithelial cells.

In conclusion, our results support the potential of *S. cerevisiae* probiotic and, likely also, inactivated yeast product as anti-infective agents in the vagina and encourage further studies about their capacity to prevent and manage urogenital tract infections in females. Our results may help devise modalities to optimize the defensive properties of the vaginal microbiota, improving the health of many women by *S. cerevisiae* administration.

## Materials and methods

### Study products

The products studied were provided by Lesaffre Human Care (Marcq-en-Baroeul, France).

*Saccharomyces cerevisiae* (*S. cerevisiae*) live yeast (referenced GI) is a proprietary, well-characterized strain of Lesaffre, registered in the French National Collection of Cultures of Microorganisms (CNCM) under the number I-3856. The *S. cerevisiae* species is characterized by using phenotypic (API®ID32C, Biomerieux SAS) and genotypic referenced methods (genetic amplification and sequencing of 26S DNA).^42,43^ Moreover, the strain CNCM I-3856 has been characterized by polymerase chain reaction (PCR) Interdelta typing techniques^44^ and other genetic methods (e.g. complete genome sequencing).

Inactivated yeast of *S. cerevisiae* (referenced IY) is a primary grown inactivated dried whole yeast, obtained by drum-drying of *S. cerevisiae*.

The concentration of GI is 1 × 10^10^ CFU/g.

### Microbial strains and growth conditions

*C. albicans* CA1398 carrying the bioluminescence *ACT1p-gLUC59* fusion product (BLI *Candida*) was used.^19,45^ The gLUC59 luciferase reporter has previously been described.^19^ The origin and the characteristics of the highly virulent *C. albicans* strain (CA-6) have previously been described.^46^ The cultures were maintained by serial passages on YPD agar. The yeast cells were harvested by suspending a single colony in saline, washed twice, counted in a hemocytometer and adjusted to the desired concentration. *Lactobacillus casei* (*L. casei*) was isolated from Italian patient and obtained from the laboratory of mycology of the Santa Maria della Misericordia Hospital, Perugia, Italy. *L. casei* was grown in Tryptic soy agar plus sheep blood.

### Ethics statement

The procedures involving the animals and their care were conducted in conformity with the national and international laws and policies. All animal experiments were performed in agreement with the EU Directive 2010/63, the European Convention for the Protection of Vertebrate Animals used for Experimental and other Scientific Purposes, and the National Law 116/92. The protocol was approved by Perugia University Ethics Committee for animal care and use (Comitato Universitario di Bioetica, permit number 149/2009-B). All the animals were housed in the animal facility of the University of Perugia (Authorization number 34/2003A). Mice were acclimatized for a week before starting the experiments. Five to eight mice were housed in each cage and were provided with food and water *ad libitum*. All efforts were made to minimize suffering during experiments.

### Mice

Female CD1 mice obtained from Charles River (Calco, Italy) were used at 4 to 6 weeks of age. Mice were allowed to rest for 1 week before the experiment; by that time the animals were roughly 5 to 7 weeks old. Animals were used under specific-pathogen free conditions that included testing sentinels for unwanted infections; according to the Federation of European Laboratory Animal Science Association standards, no infections were detected.

### Infection and treatment

Mice were vaginally infected as previously described.^19^ Mice were maintained under pseudoestrus condition by subcutaneous injection of 0.2 mg of estradiol valerate in 100 μl of sesame oil (Sigma-Aldrich) 5 d prior to infection and weekly until the completion of the study. Mice anesthetized with 2.5-3.5% (v/v) isoflurane gas were infected with 10 μl of 2 × 10^9^ cell/ml of BLI *Candida*. Cell suspensions were administered from a mechanical pipette into the vaginal lumen, close to the cervix. To favor vaginal contact and adsorption of fungal cells, mice were held head down for 1 min following inoculation. Mice were then allowed to recover for 24-48 h, during which the *Candida* infection was established. The intravaginal treatment with FLZ (200 μg/ml, 10 μl/mouse), IY (100 mg/ml, 10 μl/mouse) or GI (10 mg/ml, 10 μl/mouse) was done 1 day after the challenge or every day starting from day +1 after challenge.

### Monitoring mouse vaginal infection

At selected time points post-infection, mice were treated with 10 μl of coelenterazine (0.5 mg/ml in 1:10 methanol:H_2_O) (Synchem, OHM) in the vaginal lumen. Afterwards, mice were imaged in the IVIS-200TM imaging system (Xenogen Inc.) under anesthesia with 2.5% isoflurane. Total photon flux emission from vaginal areas within the images (Region Of Interest, ROI) of each mouse was quantified with Living ImageR software package.

### CFU assay

In selected experiments, at day +4 and +8 post-infection, the vaginal washes were conducted with 150 μl of saline, given in 3 separate 50 μl volumes. The fluid was serially diluted and plated on CHROMagar™ Candida (VWR International p.b.i., Milan, Italy). CFU were then evaluated and expressed as CFU/ml.

### Cell lines and vaginal epithelium

The human A-431 vaginal epithelial cell line and the human HeLa epithelial cervical cell line, obtained from ATCC, were grown in DMEM plus 10% of fetal calf serum (FCS). Human vaginal epithelium was obtained by the differentiation of A-431 cell line as previously described.^47^

### Adherence assay

BLI *Candida* adherence to cell lines was determined using a luminometer (Tecan). A-431 and HeLa (1 × 10^6^/ml) were grown for 24 h in black 96-well microtiter plates with a transparent bottom (100 µl/well). Vaginal epithelium was obtained by differentiation of A-431 cell line (1 × 10^6^/ml) for 5 d in black 96-well microtiter plates with a transparent bottom (100 µl/well).^47^ Before stimulation, cells were incubated for 2 h in DMEM medium without FCS. Cells were then incubated in the presence or absence of 100 µl of IY or GI (both 100 mg/ml) for 30 min or 1 h at 37°C plus 5% CO_2_, extensively washed 5 times with PBS and then incubated with 100 µl of BLI *Candida* (1 × 10^6^/ml) for 1 h. After co-incubation, non-adherent BLI *Candida* cells were removed by extensively rinsing 5 times with PBS and 100 μl of 2 μM coelenterazine in luciferase assay buffer (LA buffer) were added in each well.^19^ Luciferase activity was measured using a luminometer. Bioluminescence representative image was performed by using IVIS-200TM imaging system (Xenogen Inc.). Total photon flux emission from each well (Region Of Interest, ROI) was quantified with Living ImageR software package.

Adherence was also evaluated by colony forming units counts (CFU). To this end, the LA buffer was removed from each well, Trypsin/EDTA solution (100 µl) was added in each well, then the plates were incubated for 15 min at 37°C plus 5% CO_2_ to dissociate cells. Hence, the cellular suspension was diluted (1/10), plated onto CHROMagar Candida and incubated at 37°C for 48 h. The fungal load (the adherent BLI *Candida*) was quantified as the number of CFU/ml. In selected experiments, cells were incubated with 100 µl of IY or GI (both 100 mg/ml) as above described for 1 h, then washed 5 times with PBS and treated with Trypsin/EDTA solution (100 µl) to dissociate cells, or incubated further 18 h and then treated with Trypsin/EDTA solution (100 µl). Hence, the cellular suspension was diluted, plated onto Sabouraud agar and incubated at 37°C for 48 h.

### Fluorescent *C. albicans, L. casei*, IY and GI

CA-6 cells were harvested and the concentration was adjusted to the desired concentration and labeled with CalcoFluor White (CFW) (0.5 mg/ml) for 30 min at RT. *L. casei* (3.3 McFarland), IY and GI (10 or 100 mg/ml) were labeled with FITC at 0.1 mg/ml in PBS at RT for 10 min.^48^

### Adherence assay of FITC-IY and FITC-GI

Vaginal epithelium in black 96-well microtiter plates with a transparent bottom were incubated for 2 h in DMEM medium without FCS. 100 µl of FITC-IY or FITC-GI (both 100 mg/ml) were added in the wells and immediately analyzed for fluorescence signals (λ = 600 nm), then cells were incubated for 1 h at 37°C plus 5% CO_2_. After incubation, cells were extensively washed 5 times with PBS and then analyzed for fluorescence signals (λ = 600 nm).

### Damage assay

A-431 and HeLa cells (both 1 × 10^6^/ml) were grown for 24 h in 96-well microtiter plates (100 µl/well). Vaginal epithelium was obtained by differentiation of A-431 cell line (1 × 10^6^/ml) for 5 d in 96-well microtiter plates (100 µl/well).^47^ Before stimulation, cells were incubated for 2 h in DMEM medium without FCS. Cells were then incubated in the presence or absence of 100 µl of IY or GI (both 100 mg/ml) for 30 min or 1 h at 37°C plus 5% CO_2_, extensively washed 5 times with PBS and then incubated with 100 µl of BLI *Candida* (1 × 10^6^/ml) for 18 h at 37°C plus 5% CO_2_. After co-incubation, the epithelial cell damage was determined by the release of LDH into the surrounding medium. LDH was measured spectrophotometrically at 492 nm using a Cytotoxicity Detection kit (LDH) from Pierce (Thermo Scientific, USA). The percentage cytotoxicity of epithelial cells infected with BLI *Candida* was calculated as follows: experimental LDH release minus background cells minus background BLI *Candida* / mean maximal LDH release minus background cells and compared to 100% BLI *Candida* damage induced in each cell type.^25^ Considering maximal LDH release as 100% of damage (induced by the positive control lysis buffer), the *Candida*-induced damage to A-431 cells was 46.1%, to HeLa cells 21.0%, and to vaginal epithelium 49.2%.

### Coaggregation assay

CA-6 or CFW-CA-6 (1 × 10^9^/ml) in 500 µl of PBS were mixed with equal volume of *L. casei* or FITC-*L. casei* (3.3 McFarland), or with equal volume of IY, GI or FITC-IY and FITC-GI (all 10 mg/ml). Then the samples were vortexed for at least 10 sec and incubated in a 24 well plates for 4 h at 37°C under agitation. The suspensions were then observed by inversion light microscopy to evaluate the aggregation degree and scored according to the following scale: 0= no aggregation, 1= small aggregates comprising small visible clusters of yeasts, 2= aggregates comprising larger numbers of yeasts, settling down to the center of the well, 3= macroscopically visible clumps comprising larger groups of yeasts which settle to the center of the well, 4= maximum score allocated to describe a large, macroscopically visible clump in the center of the well.^21^ Moreover, each fluorescent suspension was analyzed under a fluorescent light microscope (Carl Zeiss).

### Germ-tube and hyphae formation

500 µl of CA-6 (1 × 10^6^/ml) was incubated in PBS or PBS + 10% FCS in the presence or absence of 500 µl of IY or GI (both 10 mg/ml) in a 24 well plate for 2 or 4 h at 37°C under agitation for evaluation of germ-tube or hyphae formation, respectively. After incubation each suspension was diluted 1/10 and then 100 µl were used for Gram stained smears, microscopically examined and counted for germ-tube and hyphae formation.^49^ In selected experiments, 500 µl of CFW-CA-6 (1 × 10^6^/ml) was incubated in PBS or PBS + 10% FCS in the presence or absence of 500 µl of FITC-IY, FITC-GI (both 10 mg/ml). After incubation each suspension was diluted 1/100 and then analyzed under a fluorescent light microscope (Carl Zeiss). Moreover, 500 µl of CA-6 (1 × 10^6^/ml) was incubated in YPD or YPD + 10% FCS in the presence or absence or 500 µl of cell-free supernatants from 24 h culture of IY and GI (both 2 mg/ml) in YPD medium, for 4 h at 37°C under agitation. After incubation each suspension was diluted 1/100 and then analyzed under a light microscope (Carl Zeiss).

### Quantitative analysis of *SAP2* and *SAP6* gene expression

CA-6 (2 × 10^6^/ml) was incubated in YEPD medium or YEPD medium plus 1% of bovine serum albumin (BSA),^24^ in the presence or absence of IY or GI (both 10 mg/ml) for 24 h at 37°C under agitation. After incubation, fungal cells were lysed using Trizol reagent (Life Technology). In selected experiments, vaginal washes from mice intravaginally infected with 10 μl of 2 × 10^9^ cell/ml of CA-6 and daily treated with IY or GI, as described above, were obtained 8 d post-infection. Vaginal washes were centrifuged at 3000 rpm for 5 min, then cellular fractions were lysed using Trizol reagent (Life Technology).

Total RNA was extracted and retro-transcribed by using the Moloney murine leukemia virus reverse transcriptase reaction (M-MLV RT), as described in the manufacturer's instructions. cDNA concentration was determined using a spectrophotometer. *C. albicans ACT1, SAP2* and *SAP6* genes were detected by using primers reported elsewhere.^50^ Real-time PCR (quantitative PCR) was performed in 96-well PCR plates using SYBR green (all from BioRad). For real-time PCR reaction 100 ng of cDNA was used. All samples were measured in triplicates and cDNA quantities reported as 2^−ΔΔCT^ relative to the *Candida* alone. Amplification conditions were the same used for *ACT1* and *SAP* genes: 3 min at 95°C, 40 cycles of 10 sec at 95°C and 30 sec at 55°C. The experiments were performed using the Eppendorf Mastercycler.

### Statistical analysis

For the analysis of fungal burden by measurement of Total photon flux emission from infected vaginal areas and CFU of vaginal washes, differences between FLZ- or IY- and GI-treated infected mice *vs* saline-treated infected mice were evaluated by Mann-Whitney U-test. Values of *p* < 0.05 were considered significant.

For the other experiments, the results reported in the bar graphs are the mean ± SEM from duplicate or triplicate samples of 3-6 different experiments. Quantitative variables were tested for normal distribution and compared by means of Student's t test. Values of *p*<0.05 were considered significant.

## Supplementary Material

KVIR_S_1213937.zip
