# Supplementary material for: Therapeutic activity of a Saccharomyces cerevisiae-based probiotic and inactivated whole yeast on vaginal candidiasis
Source: Virulence. 2016 Jul 19;8(1):74–90. doi: 10.1080/21505594.2016.1213937 (PMC5963212; doi:10.1080/21505594.2016.1213937)
Supplement: KVIR_S_1213937.zip [file kvir-08-01-1213937-s001.zip › KVIR_S_1213937.docx]

**Supplementary Figure 1. Quantification of Total photon flux emission and CFU count**. Mice under pseudoestrus condition were treated intravaginally with 10 μl of saline, FLZ (200 μg/ml, 10 μl/mouse) or different yeast products: IY (100 mg/ml, 10 μl/mouse) and GI (10 mg/ml, 10 μl/mouse), 1 day after challenge (2 x 10^7^ BLI *Candida* cells/10 μl/mouse). After 4, 6, 8, 10 and 12 days post-infection mice were treated intravaginally with 10 μl of coelenterazine (0.5 mg/ml) and imaged in the IVIS-200TM imaging system under anaesthesia with 2.5% isofluorane. Total photon flux emission from vaginal areas within the images (Region Of Interest, ROI) of each mouse was quantified with Living ImageR software package. Quantification of Total photon flux emission from ROI (n=10 mice for each group from 2 different experiments) was evaluated and the graphs show the % of Total photon flux inhibition of the treatments (compared to 100% saline-treated infected mice). For details about statistical significant difference between experimental groups see Figure 2.

**Supplementary Figure 2. Quantification of Total photon flux emission and CFU count.** Mice under pseudoestrus condition were treated intravaginally with 10 μl of saline, FLZ (200 μg/ml, 10 μl/mouse) or different yeast products: IY (100 mg/ml, 10 μl/mouse) and GI (10 mg/ml, 10 μl/mouse), every day starting from day +1 after challenge (2 x 10^7^ BLI *Candida* cells/10 μl/mouse). After 4, 6, 8, 10 and 12 days post-infection mice were treated intravaginally with 10 μl of coelenterazine (0.5 mg/ml) and imaged in the IVIS-200TM imaging system under anaesthesia with 2.5% isofluorane. Total photon flux emission from vaginal areas within the images (Region Of Interest, ROI) of each mouse was quantified with Living ImageR software package. Quantification of Total photon flux emission from ROI (n=10 mice for each group from 2 different experiments) was evaluated and the graphs show the % of Total photon flux inhibition of the treatments (compared to 100% saline-treated infected mice). For details about statistical significant difference between experimental groups see Figure 4.
