# Supplementary figures and images for: Therapeutic activity of a Saccharomyces cerevisiae-based probiotic and inactivated whole yeast on vaginal candidiasis
Source: Virulence. 2016 Jul 19;8(1):74–90. doi: 10.1080/21505594.2016.1213937 (PMC5963212; doi:10.1080/21505594.2016.1213937)

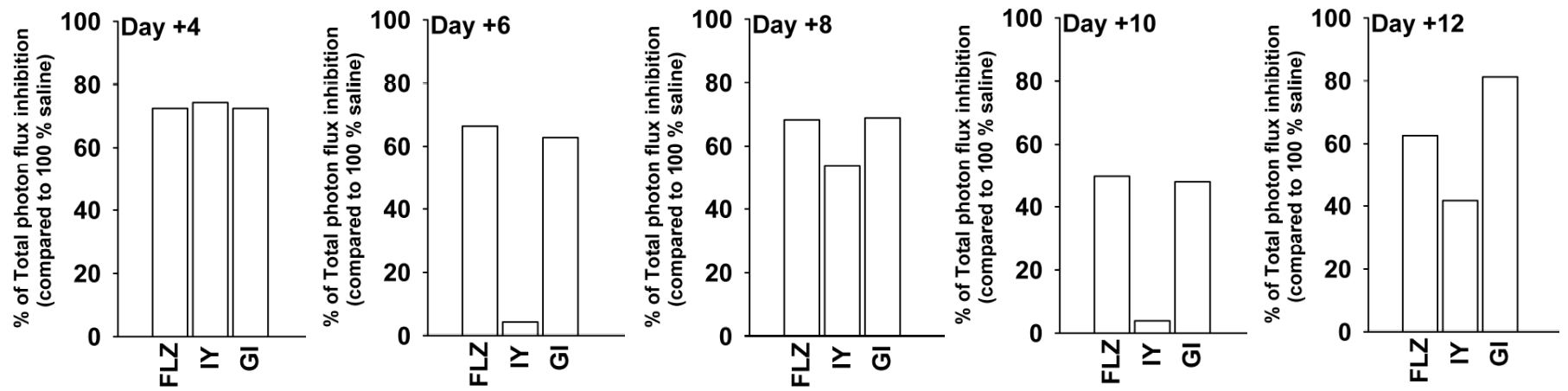

Supplementary Figure 1

Supplement: KVIR_S_1213937.zip [file kvir-08-01-1213937-s001.zip › KVIR_S_1213937_Fig 1.pdf]

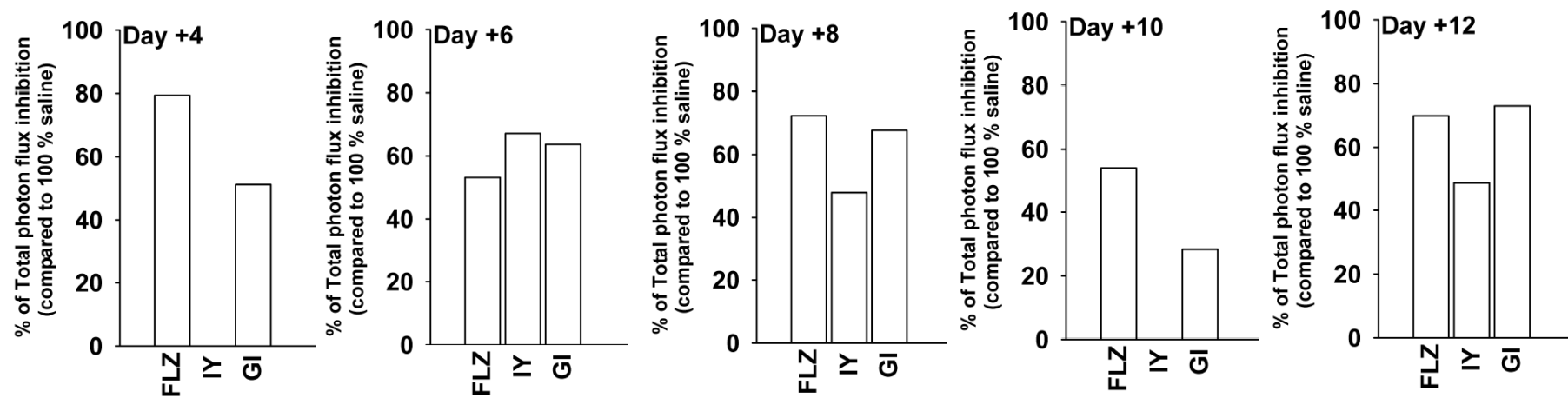

Supplementary Figure 2

Supplement: KVIR_S_1213937.zip [file kvir-08-01-1213937-s001.zip › KVIR_S_1213937_Fig 2.pdf]
